# You Seem Certain but You Were Wrong Before: Developmental Change in Preschoolers’ Relative Trust in Accurate versus Confident Speakers

**DOI:** 10.1371/journal.pone.0108308

**Published:** 2014-09-25

**Authors:** Patricia Brosseau-Liard, Tracy Cassels, Susan Birch

**Affiliations:** 1 Department of Psychology, Concordia University, Montreal, Quebec, Canada; 2 Department of Psychology, University of British Columbia, Vancouver, British Columbia, Canada; Birkbeck, University of London, United Kingdom

## Abstract

The present study tested how preschoolers weigh two important cues to a person’s credibility, namely prior accuracy and confidence, when deciding what to learn and believe. Four- and 5-year-olds (*N* = 96) preferred to believe information provided by a confident rather than hesitant individual; however, when confidence conflicted with accuracy, preschoolers increasingly favored information from the previously accurate but hesitant individual as they aged. These findings reveal an important developmental progression in how children use others’ confidence and prior accuracy to shape what they learn and provide a window into children’s developing social cognition, scepticism, and critical thinking.

## Introduction

Young children have a lot to learn about the world around them. Much of this information must be learned from others because some information is impossible to learn without social input (e.g., words are arbitrary social conventions); other information is impractical, inefficient, or extremely difficult to learn on one’s own (e.g., the location of one’s kidneys or distance between two planets); and still other information is too dangerous to learn on one’s own (e.g., what animals are safe to touch). Unfortunately, learning from others comes with its own major challenge: People can and do misinform others, whether out of ill-will or because they are ignorant, misinformed, or biased. Consequently, it is paramount for learners to be critical consumers of information rather than passively accepting all information others provide.

Fortunately, children capitalize on a number of cues to increase their chances of learning accurate and self-relevant information, including age [1]–[2], familiarity [3], or group membership [4]. There are a variety of cues children can use to determine whether a speaker is likely knowledgeable about the information that they convey. One can, for instance, recall an individual’s past accuracy: All else being equal, an individual who has frequently been accurate in the past is a safer bet to learn from than an individual who has frequently provided false information in the past. A plethora of recent studies have demonstrated that toddlers and preschool children prefer to learn from previously accurate rather than previously inaccurate individuals [5]–[10]. Another cue that children can use to evaluate an individual’s knowledge is the level of confidence or certainty they express when providing information. Studies have demonstrated that young children attend to several confidence markers. For example, preschoolers are sensitive to the difference between “know” and “guess” [11] and are more likely to trust information coming from someone who claims to “know” than from someone who verbally expresses uncertainty [12]–[13]. Importantly, children as young as age 2 are also attentive to nonverbal markers of confidence and uncertainty, being more likely to imitate someone’s actions if that person appeared confident in her body language rather than uncertain [14]–[15].

Although past studies have shown that children can use cues such as a model’s prior accuracy and level of confidence to guide their selective social learning, little is known about how children weigh these two cues and what they understand about the relative utility of these cues for predicting others’ knowledge. There have been many recent studies examining how children weigh different credibility cues when more than one cue is available. Though preschoolers prefer to learn from previously accurate individuals over previously inaccurate ones, this preference can disappear or even be reversed if the individuals also vary on other knowledge indicators. For instance, when choosing to learn about the contents of a box from either a previously accurate individual who has not looked inside the box or a previously inaccurate individual who has looked inside, 5-year-olds select the individual who looked inside, regardless of past accuracy^16^. In addition, several studies have examined how preschoolers weigh age differences with other potential knowledge indicators. Overall, most studies find that young children’s preference to learn from older individuals is overridden by cues such as prior accuracy [1], expertise [17], or perceptual access [18] (though not all studies show this pattern [19]). Other attributes, such as familiarity [3] or similarity [20], can influence children’s propensity to selectively learn from an individual; the relative impact of these cues in relation to a different cue, such as prior accuracy, varies during the preschool years.

Since both confidence and accuracy are cues that can be informative about an individual’s knowledge, it is important to see how children weigh these two cues and whether the weight granted to these cues varies during the preschool years. Confidence can be a useful predictor of knowledge in a given situation at a given point in time, however, it is a *subjective* assessment by the person of their own knowledge and therefore not always correlated with the actual credibility of their information [21]. There are also important individual differences in people’s likelihood of expressing confidence or uncertainty (such as differences in personality styles) – differences that are not necessarily related to underlying differences in knowledge. In contrast, an individual’s past accuracy is a more objective measure of the individual’s level of knowledge. Though it is very possible that someone who has been accurate in the past might subsequently provide inaccurate information (and vice versa), past accuracy tends to correlate with current and future knowledge and at least provides a measure of an individual’s credibility that is independent of that individual’s subjective assessment of his or her knowledge. Children’s ability to appreciate these distinctions when making decisions of who to believe, and when, will have a profound impact on what they learn about the world. Although not all cases will be so extreme, to illustrate the importance of this ability imagine the dire consequences for a child who is persuaded by a sibling’s confident claims that a wild mushroom (or other toxic substance) is “really good” despite the sibling’s history of making false assertions.

To our knowledge, only one study so far has investigated children’s weighting of confidence and accuracy cues [22]. These researchers were interested in studying differences between children and adults’ ability to use *calibration*, or the congruence between individuals’ confidence and their accuracy, when judging the credibility of mock eye-witness testimony. Both adult participants and 5- to 6-year-old children heard a story involving an accident, where two “witnesses” provided testimony about features of that event. Both witnesses were accurate once and inaccurate once. One of the witnesses (witness A) was poorly calibrated: She was confident in both cases, regardless of her accuracy. The other witness (witness B) was better calibrated: She was confident when she made the correct statement and unconfident when she made a statement that later turned out to be incorrect. The two witnesses then provided more facts, always with confidence, and participants were asked which witness they believed. The adults, when not under cognitive load, later chose the well-calibrated witness as the reliable source, whereas the five- and six-year-olds (and the adults under cognitive load) relied on the consistently confident, but poorly calibrated, witness. In other words, adults who were not under cognitive load appeared to understand that the speaker’s level of confidence was a better predictor of accuracy for witness B (i.e., she at least appeared aware of her own uncertainty when providing an inaccurate fact); children, however, appeared to rely solely on the witnesses’ level of confidence when deciding who to believe.

Does this mean that young children grant excessive weight to confidence and fail to notice when confidence is unwarranted? Arguably, a prerequisite for understanding calibration is an appreciation of the differential informativeness of the two component cues. In the present manuscript, we investigate how young children weigh consistent individual differences in accuracy and confidence. That is, we investigated whether children appreciate these prerequisites for understanding calibration. We aimed to answer two specific questions: First, are children more likely to use confidence or past accuracy to decide whom to learn from if the two cues are in conflict? Second, are there developmental changes in children’s weighting of these cues over the preschool years?

To test this question, we presented children with a learning situation where information about a source’s past accuracy conflicted with their expressed confidence. If children grant the greatest weight to confidence, they should believe confident individuals regardless of their prior accuracy. If, however, children view past accuracy as a better indicator of knowledge than confidence, they should preferentially learn from a *hesitant* individual when that individual has previously demonstrated greater accuracy.

## Methods

### Participants

We tested 96 four- and 5-year-olds (ages: 4, 0–5, 11; *M* = 5, 0; 42 females). Children were recruited from a local science museum and a database of interested families. Though demographic information was not systematically collected, the sample included diverse families in terms of race, ethnicity and socioeconomic status. Three additional children were tested but their data were excluded either because they failed to answer all test questions (1) or their parents did not provide their birthdate (2). The use of human subjects for this research was approved by the University of British Columbia’s Behavioural Research Ethics Board. Written consent was obtained from participants’ parents, and child participants verbally agreed to participate before the beginning of the study.

### Materials

A series of short videoclips showing two adult females talking about a variety of animals were presented on a computer screen. These were accompanied with paper pictures showing the different animals as well as printed pictures of the two adults.

### Procedure

Children were seated in front of a computer screen. An experimenter explained to the child that they would hear facts about animals from two people shown on video. Children were shown two short videoclips introducing the two informants, and two pictures depicting the informants were placed in front of the child to help them remember their identity. The informants were always presented in the same order but which informant served as the confidently inaccurate informant was counterbalanced. After the introduction of the informants, children in the Confidence + Accuracy condition were presented with a History Phase (described below). Children in both the Confidence + Accuracy condition and Confidence Only condition (who did not receive a history phase) were presented with the Test Phase (described below). Half of the children took part in the Confidence + Accuracy condition and the other half took part in the Confidence Only condition.

#### History Phase

Children in the Confidence + Accuracy condition saw four history trials, each involving one picture of an animal that is familiar to young children (a whale, a duck, a cow and a frog). For each picture, the experimenter told the child that the two informants (Nena and Joyce) would tell them about the animal. For instance, for the whale, the experimenter said: “Let’s hear what Nena and Joyce say about where whales live.” The experimenter then played two videos. One clip showed one of the informants stating an accurate fact (e.g., “whales live in the water”) but with cues of hesitancy, including verbal (i.e., saying “I guess”), paralinguistic (e.g., upward inflexion) and non-verbal cues (e.g., puzzled facial expressions, shrugging her shoulders); and the other clip showed the other informant stating an inaccurate fact (e.g., “whales live in the ground”) but with cues of confidence, including verbal (i.e., saying “Oh, I know!”), paralinguistic (e.g., declarative tone) and non-verbal cues (e.g., satisfied facial expression, raised index finger). This procedure was repeated for 4 trials, with the same informant being consistently accurate but hesitant and the other informant being consistently inaccurate but confident. Nena’s videos were always shown before Joyce’s, however who was hesitant and who was confident was alternated between participants.

#### Test Phase

Children in both conditions were presented with four test trials, each involving one picture of a type of animal that is unfamiliar to most children (i.e., a lanternfish, a Philippine eagle, an Iberian lynx and a pygmy sloth). The experimenter told children that they would hear the informants tell them facts about the animals and that they would then be asked what they thought. On each trial, the experimenter stated that they would hear the informants provide a specific fact (e.g., “Let’s hear what Nena and Joyce say about what that fish is called”). The experimenter then played two videos: One clip showed one of the informants stating one fact (e.g., “it’s called a lanternfish”) with cues of hesitancy, and the other clip showed the other informant stating a conflicting fact (e.g., “it’s called a paddlefish”) with cues of confidence. For those children in the Confidence + Accuracy condition, the same informant who had previously been confident remained confident on test trials and the one who had been hesitant remained hesitant. The identity of the confident source (Nena vs. Joyce) alternated between participants, as did the set of answers provided by each individual. After each pair of videos, children were asked what they thought (e.g., “What do you think? Do you think it’s called a lanternfish or a paddlefish?”). If they did not spontaneously answer, the experimenter first prompted them to guess, and if children still did not answer, she gave them the opportunity to point to the informants’ pictures rather than provide a verbal answer (e.g., “Do you think it’s a lanternfish like she said [pointing to the picture of the informant who said *lanternfish*] or a paddlefish like she said [pointing to the picture of the informant who said *paddlefish*]? Can you point?”).

#### Post-Test Questions

After all four trials, children were asked three post-test questions. The first two questions checked whether children could accurately state which informant had provided which fact on the last test question to ensure they were paying attention. The third question varied by condition. Those in the Confidence + Accuracy condition were asked a question testing their memory of the history phase to ensure they recognized one informant as more accurate than the other. They were shown the pictures of the four familiar animals, and were told “One of my friends said things that were right about these animals, and the other one said things that were wrong. Which one was right?”. Those in the Confidence Only condition, who were not presented with the history phase, were instead asked which informant they thought was “smarter” to ensure they perceived the difference between the two informants.

## Results

The number of times out of 4 trials in which children chose the same answer as the confident individual served as the dependent variable. Planned t-tests revealed that preschoolers were overall more likely than chance to trust the confident individual in the Confidence Only condition (*M* = 2.35 trials or 58.8%; *t*(47) = 2.19, *p* = .033, *d* = .31), consistent with previous research. In the Confidence + Accuracy condition, however, preschoolers were significantly *less* likely than chance to trust the confident (and inaccurate) individual (*M* = 1.62 out of 4 trials or 40.5%; *t*(47) = 2.03, *p* = .048, *d* = .30).

**Figure 1:**
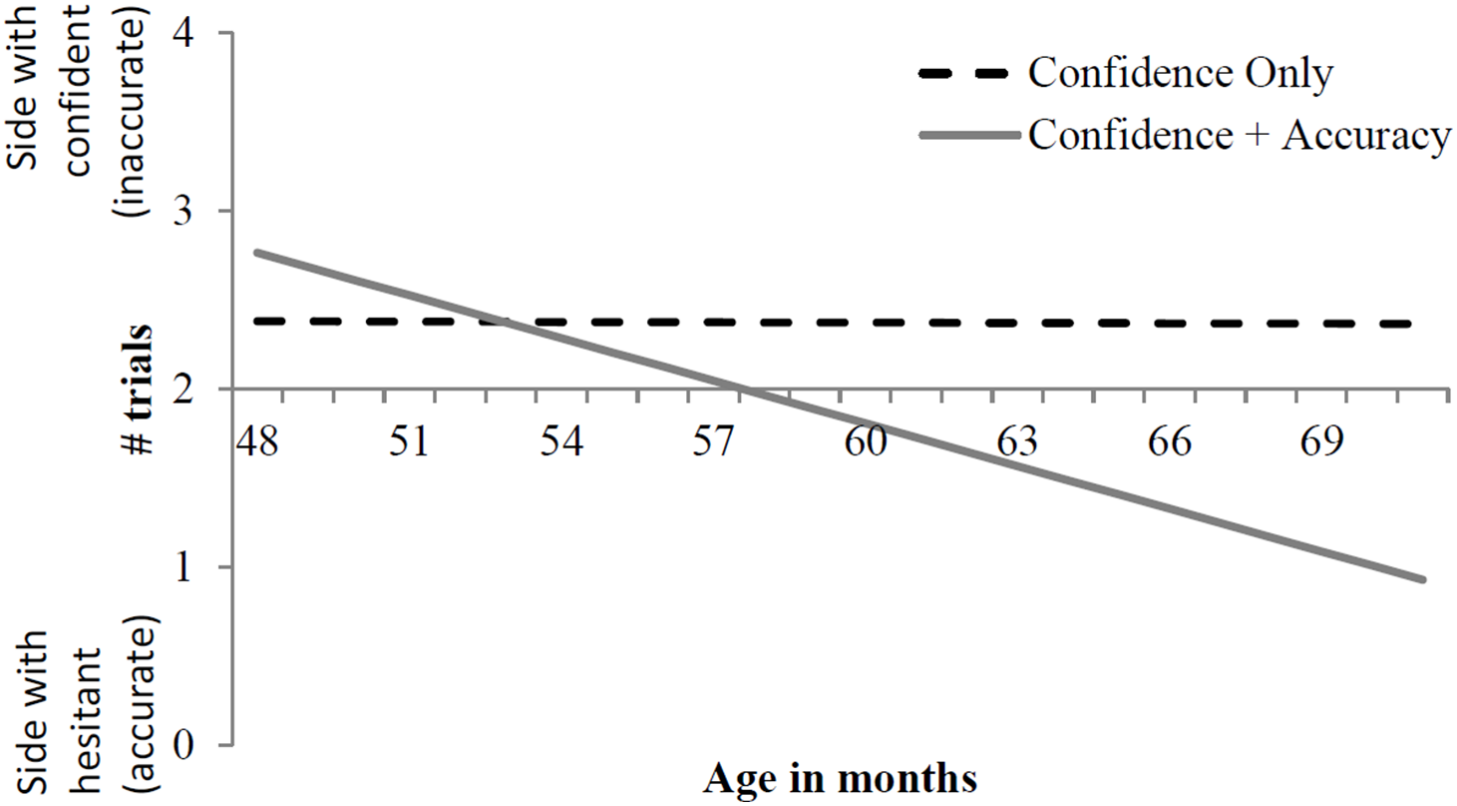
Simple slopes for predicted propensity to side with the confident individual by age.

Preliminary analyses ruled out any main effect or interaction involving sex or the identity of the confident individual, but revealed a significant main effect of Answer Set (though, importantly, no interactions involving Answer Set). We conducted a multiple regression predicting the propensity to side with the confident individual with the following predictors: Condition (Confidence only or Confidence + Accuracy), Age (in months), Answer Set, and an interaction term between Condition and Age. Children’s propensity to side with the confident individual varied significantly based on Condition, β = .253, *p* = .006; Age, β = .210, *p* = .022; and Answer Set, β = .335, *p*<.001. The interaction between Age and Condition was significant, β = .208, *p* = .024. We further explored this interaction by examining simple slopes for age as a function of condition (see Figure 1). In the Confidence Only condition, children at the mean age of our sample (5 years 0 months) sided with the confident individual on 2.37 out of 4 trials, and this propensity did not vary significantly by age (a non-significant decrease of less than .001 trials for each 1-month increase in age). In the Confidence + Accuracy condition, children at the mean age of our sample sided with the confident (but inaccurate) individual on 1.74 out of 4 trials, but this propensity significantly decreased by.08 for each 1-month increase in age (*p* = .002).

To further illustrate the age-related change in the Confidence + Accuracy condition, we performed a median split on age and conducting one-sample *t*-tests comparing each resulting group’s mean number of trials trusting the confident but previously inaccurate informant against chance. Younger children (4, 1–5,1; *N* = 24) were not different from chance (*M = *1.96 or 49%; *t*(23) = .16, *ns*). In contrast, older children (5, 2–5,11; *N* = 24) were significantly below chance in their propensity to trust the confident individual (and therefore above chance at trusting the hesitant but accurate individual); *M* = 1.29 trials or 32%, *t*(23) = −2.90, *p* = .008, *d* = .59.

The significant effect of Answer Set was unexpected: Children were more likely to endorse the second set of answers than the first set. Importantly, however, our preliminary analyses had not revealed any significant interaction between Answer Set and Condition. The apparent preference for one of the answer sets was not of theoretical interest; still, we performed additional analyses to ensure that the patterns of results were similar across answer sets. Indeed, they were: We performed independent-samples t-tests looking at the effect of Condition separately for children in each Answer Set condition. Note that this splits the sample and therefore reduces our power to detect small effects. The effect of Condition was significant for children who heard the confident individual provide the second set of answers, *t*(42) = 2.48, *p* = .012, *d* = .74, and was marginally significant for the first set, *t*(50) = 1.76, *p* = .085, *d* = .49. We also examined the correlation between age in months and children’s responses separately in each Answer Set condition. In the Confidence Only condition, children’s age did not correlate significantly with propensity to endorse the confident individual’s responses in either Answer Set condition (first set: *r*(25) = .020, *ns*; second set: *r*(23) = −.001, *ns*). In contrast, children’s propensity to trust the confident source decreased with age in the Confidence + Accuracy condition for both answer sets, though the correlation was only significant for one of the answer sets (first set: *r*(27) = −.440, *p* = .022; second set: *r*(21) = −.361, *p* = .108, *ns*).

Children’s responses on post-test questions showed that they could use both cues to discriminate between the two informants. In the Confidence Only condition, 37 out of 48 children (77%) identified the confident individual as “smarter” (binomial *p*<.001). In the Confidence + Accuracy condition, 35 out of 48 children correctly identified the informant who had provided accurate information (binomial *p* = .002). Their success at answering this post-test question allows us to rule out the possibility that younger children’s failure to systematically favour accuracy over confidence was simply because they forgot or did not notice which individual had been accurate. There did not seem to be any age effects on the memory question: the children who failed the post-test question in the Confidence + Accuracy condition were almost evenly distributed between younger (*N* = 8) and older children (*N* = 5) using the median split criteria. Furthermore, the correlation between age and propensity to trust the hesitant but accurate informant remained (and if anything got even stronger) when removing children who failed the post-test memory question (*r*(35) = −.511, *p* = .002). Thus, memory demands are unlikely to account for the younger children’s lack of a clear preference between a confident but inaccurate individual and a hesitant but accurate one.

## Discussion

We demonstrated that preschool children are sensitive to the confidence of others when deciding what to believe; importantly, however, with increasing age, they become more likely to favor the information provided by a hesitant individual who has demonstrated greater prior accuracy (over that of a confident individual who has a history of inaccuracy).

What accounts for these developmental changes? There are a few possible explanations to consider. First, it is possible that, although younger preschoolers understand that a) a confident individual is more likely knowledgeable than a hesitant one (or at least they are inclined to favor information from a more confident source) and b) that a previously accurate individual is more trustworthy than a previously inaccurate one (or at least they are inclined to favor information from a more accurate source), they nonetheless lack an appreciation of which is the better indicator of knowledge, and therefore perform at chance when the two cues conflict. Older preschoolers, in contrast, may favour past accuracy because they see it as a more important, more objective, or simply more salient indicator of knowledge (though without necessarily being able to explicitly recognize or verbally express these reasons). Under this first explanation then, the developmental changes we observed reflect a developmental progression in children’s understanding of the *relative* informativeness of the two cues (i.e., their understanding of which is the better of the two cues). Another possibility is that both younger and older preschoolers see past accuracy as a more important knowledge cue than confidence, but that younger children have a more difficult time inhibiting the readily-observable confidence cues and are therefore more likely to be swayed by these cues than older children. Under this possibility, then, the developmental change is not in the *understanding* of the relative importance of the cues but instead in the *application* of this understanding. This latter notion may be most consistent with past findings showing that even adults struggle at integrating knowledge cues when under cognitive load^21^. Future research could attempt to tease apart these possible explanations.

Note that, in the present study, the two conditions were asymmetrical, in that only one condition involved a History Phase. This was done intentionally to ensure that children in the Confidence Only condition did not have any information about the individuals except their level of confidence as they were providing novel information – our goal in that condition was strictly to test the effect of an individual’s confidence on children’s propensity to side with that individual. If the individuals had provided any known accurate (or inaccurate) information before the test phase, this would have provided children with extra information about the individuals’ value as sources of information and potentially changed their propensity to side with each individual. If the individuals had provided novel information before the test phase, the amount of novel information provided to the children would have been asymmetrical between the conditions, potentially resulting in greater processing demands in the Confidence Only than the Confidence + Accuracy condition. It was thus unavoidable to have an unequal amount of information between the two conditions; the fact that children in the Confidence + Accuracy condition generally passed the memory question and that the correlation between age and propensity to side with each individual remained even when removing children who failed the memory question suggests that processing demands are not solely responsible for the findings in that condition. In addition, note that the history phase was quite short (around 5 minutes) and did not require any demands on the part of the participants except watching and listening.

The present study reveals a more sophisticated understanding of these knowledge cues at a younger age than a past study [22] in which 5- and 6-year-olds struggled to use information about the calibration of an individual’s confidence and accuracy to moderate their social learning and instead relied on between-individual differences in confidence. Contrary to this past work, we show that by age 5 children can appropriately discount an inaccurate individual’s confidence or an accurate individual’s hesitancy. Note that the present study did not require children to process within-individual variations in confidence and accuracy, but only differences between individuals. As we argued above, tracking the covariation between an individual’s confidence and the accuracy of their different statements is a complex task, which even adults struggle with when under cognitive load. This suggests that processing demands might be an important factor in children’s more simplistic use of the cues. Tracking an individual’s calibration would seem to be a fairly complex task: Individuals’ accuracy and confidence vary from statement to statement, requiring the tracking of the covariation between these cues *within* an individual in addition to differences in this covariation *between* individuals. Our results suggest that young children do not necessarily always favor confidence over past accuracy and in fact become increasingly sceptical of overconfidence as they get older.

The present research adds to the literature on how children weigh different cues to choose the most credible source of information. Past research has shown that children can use various indicators of confidence to moderate their social learning [12], [14]–[15] and also prefer to learn from previously accurate rather than previously inaccurate individuals [5], [23]. However, these cues are often not present in isolation in the real world, and some cues are more important than others to guide one’s selective learning. As reviewed above, past research has demonstrated that preschoolers grant greater weight to past accuracy over age [1], favour perceptual access over age [18] and, in some cases, perceptual access over past accuracy [16]. Between the ages of 3 and 5 children become increasingly likely to trust an unfamiliar but recently accurate individual over a familiar but recently inaccurate one [3]. However, 5-year-olds are also more likely than younger preschoolers to trust an *inaccurate* individual who is more similar to themselves than an accurate but dissimilar individual [20]. The present study is consistent with much of this prior research showing that especially older preschoolers have a relatively sophisticated understanding of the relative value of knowledge cues; yet it provides an important addition to this prior research by showing a developmental progression in the preschool years in how children weigh the knowledge cues of an informant’s confidence and prior accuracy when deciding what information to believe.

Future research could explore how children weigh different *aspects* of confidence. For instance, in the present work we presented children with individuals who displayed verbal, nonverbal, and paralinguistic cues of confidence, to maximize the likelihood that children would pick up on at least some of the confidence cues. Future work could investigate which of these indicators of confidence children attend to most, and under which circumstances. Additionally, confidence can vary between individuals (i.e., some individuals always sound more confident than others) or within an individual across situations (i.e., one is more likely to sound confident when one is actually more certain). If someone who is habitually cautious happens to express great confidence in a specific situation, it may be wise to grant more weight to this confidence than to the same level of confidence provided by someone who is consistently overconfident.

In summary, the present study suggests that around the time of their fifth birthday children appropriately grant greater weight to someone’s prior reliability over that person’s current level of confidence. This form of emerging skepticism will serve them well as they navigate through a world selecting ‘better’ from ‘worse’ sources of information.
